# Dynamic Changes in Single Unit Activity and Gamma Oscillations in a Thalamocortical Circuit during Rapid Instrumental Learning

**DOI:** 10.1371/journal.pone.0050578

**Published:** 2012-11-30

**Authors:** Chunxiu Yu, David Fan, Alberto Lopez, Henry H. Yin

**Affiliations:** 1 Department of Psychology and Neuroscience, Duke University, Durham, North Carolina, United States of America; 2 Department of Neurobiology, Duke University, Durham, North Carolina, United States of America; 3 Center for Cognitive Neuroscience, Duke University, Durham, North Carolina, United States of America; University of British Columbia, Canada

## Abstract

The medial prefrontal cortex (mPFC) and mediodorsal thalamus (MD) together form a thalamocortical circuit that has been implicated in the learning and production of goal-directed actions. In this study we measured neural activity in both regions simultaneously, as rats learned to press a lever to earn food rewards. In both MD and mPFC, instrumental learning was accompanied by dramatic changes in the firing patterns of the neurons, in particular the rapid emergence of single-unit neural activity reflecting the completion of the action and reward delivery. In addition, we observed distinct patterns of changes in the oscillatory LFP response in MD and mPFC. With learning, there was a significant increase in theta band oscillations (6–10 Hz) in the MD, but not in the mPFC. By contrast, gamma band oscillations (40–55 Hz) increased in the mPFC, but not in the MD. Coherence between these two regions also changed with learning: gamma coherence in relation to reward delivery increased, whereas theta coherence did not. Together these results suggest that, as rats learned the instrumental contingency between action and outcome, the emergence of task related neural activity is accompanied by enhanced functional interaction between MD and mPFC in response to the reward feedback.

## Introduction

Extensive evidence implicates the prefrontal cortex (PFC) in the organization of goal-directed behavior [1], [2], [3]. But its functional interaction with other brain regions remains poorly understood. In particular, the mediodorsal thalamus (MD), a structure with extensive reciprocal connections with the mPFC, has been implicated in the learning of goal-directed actions [4], [5], [6], [7], [8], [9], [10], [11], [12], [13], [14]. MD and PFC are critical components in both the associative and limbic thalamocortico-basal ganglia networks [15], [16], [17], [18], [19], [20].

Previous lesion studies have implicated both the MD and medial prefrontal cortex (mPFC) in the acquisition and performance of reward-guided actions. MD lesions impaired learning of stimulus-reward associations [21], [22], [23] and action-reward associations [4], [24], [25]. Lesions of the mPFC lesions can also produce similar effects [2], [4], [26], [27], [28], [29], [30].

However, despite their well established anatomical connectivity, the functional interaction between MD and mPFC during goal-directed behavior remains poorly understood, because no previous study has recorded activity from both regions simultaneously during goal directed behavior. Based on previous work [4], [28], we hypothesized that instrumental learning is accompanied by significant changes in the coordination of medial prefrontal and mediodorsal thalamic activity. We predicted that, as rats learn to perform reward-guided actions, activity in both regions will change to reflect the acquisition of the action-outcome instrumental contingency. To test this hypothesis, we chronically implanted miniaturized multi-electrode arrays (up to 64 channels) in rats to record from the MD and mPFC as they learned to press a lever to earn rewards. We recorded single unit activity as well as local field potential (LFP) chronically in both MD and mPFC as rats were trained to press a lever for food reward. We measured the oscillatory activity in these brain regions simultaneously across successive days of instrumental learning. Our results show that, in the MD-mPFC circuit, dynamic changes in both single unit spiking activity and oscillatory LFP response in neuronal populations accompany the learning of a new action.

## Materials and Methods

### Ethics Statement

All procedures were approved by the Institutional Animal Care and Use Committee at Duke University and followed National Institutes of Health guidelines (Protocol Number: A087-08-03).

### Animals and Surgery

**Figure 1:**
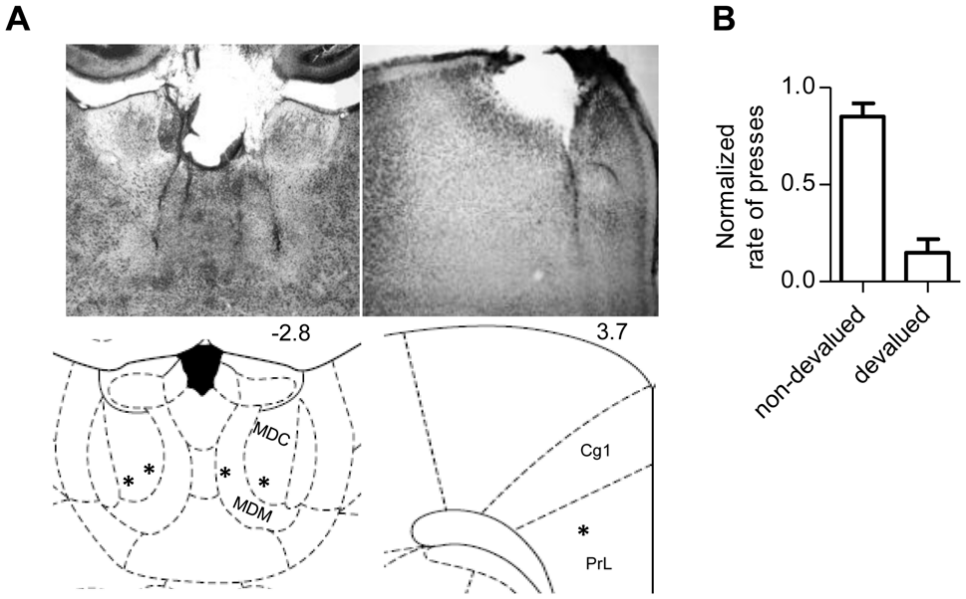
Electrode placement and behavioral results. A, Coronal sections of the rat brain illustrating MD and mPFC electrode placements. The coordinates are based on a standard rat brain atlas [58]. The numbers indicate distance in mm from Bregma. MDC, mediodorsal thalamic nucleus, central part; MDM, mediodorsal thalamic nucleus, medial part; Cg1, cingulate cortex, area1; PrL; prelimbic cortex. B, Outcome devaluation test. Devalued, rats received 1 h of unlimited food pellets, same as earned by lever pressing. Non-devalued, rats did not receive any food for 1 h before test. Normalized rate of presses were the ratio of presses under each condition. Error bars indicate SEM.

Eight male Long-Evans rats (∼3 months of age at the beginning of the experiments) were used: in 5 rats we recorded single unit and LFP activity from MD and mPFC simultaneously, and in 3 rats we recorded from MD only. Surgery was performed under general anesthesia with isoflurane (2%). A craniotomy was performed over the bilateral thalamic and/or cortical locations according to known stereotaxic coordinates (from bregma in mm the coordinates were MD AP -2.1–3.3; ML-1-1; mPFC AP 4.6–2.5; ML -1-1). The electrode arrays used in this study consisted of 4×8 or 2×8 platinum-coated tungsten microwire electrodes (35 µm diameter, Innovative Neurophysiology, NC), with 150 µm between microwires, and 200 µm between rows. The arrays were lowered to the appropriate stereotaxic depth (MD ∼5.0 mm, mPFC ∼2.5 mm,). Electrode placement was confirmed post-mortem after perfusion and fixation with 10% formalin, followed by Thionin staining in 100 µm coronal sections (Figure 1).

### *In vivo* Multi-electrode Recording during Instrumental Learning

Two weeks after surgery, rats were food deprived and maintained at ∼85% of free feeding weight throughout the experiments. Training took place in a Med Associates (St. Albans, VT) operant chamber designed for *in vivo* extracellular recording. The chamber was equipped with a food magazine that received 45 mg dustless precision pellets (Bio-Serv, NJ) from a pellet dispenser and two retractable levers on either side of the magazine and a 3 W 24 V house light mounted on the wall opposite the levers and magazine. A computer with the Med-PC-IV program was used to control the equipment and record behavior. Time stamps for lever pressing behavior and reward delivery were sent as TTL pulses to the Blackrock Cerebrus data acquisition system.

Lever press training consisted of four daily sessions under a continuous reinforcement schedule (CRF, each press earns one food pellet). Each session started with illumination of the house light and insertion of the lever, and ended with turning off the house light and retraction of the lever after 120 minutes or 100 earned pellets (whichever came first). The amount of training used was based on previous work on instrumental conditioning, which showed that performance was goal-directed following limited training [31]. In a pilot experiment, we also verified the goal-directed control of the instrumental performance using an outcome devaluation procedure. Rats (n = 4) were given a 90-min pre-feeding session using the same pellets as the training sessions. They were then tested on a 2-min probe test conducted in extinction, i.e. without any reward delivery.

Single-unit and LFP activity were recorded using the Cerebrus data acquisition system (Blackrock Microsystems). For 4×8 electrodes arrays, a TBSI (Triangle Biosystems) gain 2 headstage were used. For 2×8 arrays, the Blackrock gain 1 headstage were used, as recently described [32], [33]. In brief, the data were filtered with both analog and digital bandpass filters (analog high-pass first order Butterworth filter at 0.3 Hz, analog low-pass third order Butterworth filter at 7.5 kHz) and sampled at 30 kHz. Single unit data was separated with a high-pass digital filter (fourth order Butterworth filter at 250 Hz), while local field potential (LFP) signals were filtered with a third order high-pass filter and seventh order low-pass filter (0.1 Hz–5 Hz cutoffs).

Spikes were sorted using Offline Sorter (Plexon) and single-unit activity was isolated on the basis of principal component analysis. Only single-unit activity with a clear separation from noise was used for the analysis. Matlab was used to remove 60 Hz line noise and large transient artifacts in the LFP data: 60 Hz noise was removed using a blocked least mean squares (LMS) adaptive filter algorithm. The reference signal for the adaptive filter was created by finding the peak frequency of the LFP signal near the expected line noise frequency, and creating a sinusoidal reference signal with that frequency. The step size of the LMS algorithm was estimated by running the algorithm on a portion of the input signal for a range of varying step sizes, and using the step size that yielded the lowest RMS value of the error. Large transient motion artifacts were removed by subtracting a 20-sample moving window average around portions of the line-noise filtered signal with amplitude of greater than 6 standard deviations from the mean.

### Data Analysis

Neuronal data analysis was performed with Neuroexplorer (Nex Technologies), Microsoft Excel, Graphpad Prism (GraphPad Software), and MATLAB (MathWorks). Neural activity was averaged in 50-ms bins, averaged across trials, and smoothed with a Gaussian filter to construct the Peri-Event histogram. To classify “action initiation” neurons, neural activity within 500 ms before the onset of lever pressing was compared to a baseline window from 1500 ms to 1000 ms before the lever press (two tailed t test was used, p<0.01). To classify "reward delivery" neurons, neural activity within a 1000 ms window after reward delivery was compared with a baseline window from 2000 to 1000 ms before reward delivery. The time windows used were based on visual inspection of the data.

Spectral analysis of LFP power and coherence was performed by using Neuroexplorer. The power spectra were calculated using Welch’s method (512 frequencies between 1 and 100 Hz, smoothed with a Gaussian Kernel with bin width 3). Coherence is a measure of the linear correlation between two signals as a function of frequency [34], [35]. Coherence between two signals is calculated by dividing the cross-spectral density function by the auto –spectral density function. The cross spectrum between two time series and the auto-spectrum of each signal are obtained by calculating the product of the Fast Fourier transformed series. The signals are then subdivided into time intervals of length equal to the number of frequency samples divided by the maximum frequency, and the spectra are estimated by averaging the spectrum over these intervals (Welch’s method). The coherence measure is sensitive to both a change in power and a change in phase relationships. Consequently, if either power or phase changes in one of the signals, the coherence value is affected. In our study, Coherence analysis between LFPs from two regions was performed using 512 frequencies between 1 and 100 hz with a 5% overlap window, smoothed with a Gaussian kernel with bin width = 3.

## Results

### Behavior

All rats were naive when training began. Within the very first session of training, they learned to press the lever for reward, and their performance improved over 4 days. Previous work has established that with such limited training, instrumental behavior is highly goal-directed, sensitive to devaluation of the outcome 31,36. In a separate experiment, we assessed the effect of outcome devaluation on lever pressing with limited training. After the same amount of CRF training, rats were given 1 hour of exposure to unlimited amount of food pellets just before a 2-min probe session conducted in extinction. Outcome devaluation by pre-feeding significantly reduced instrumental performance (n = 4, paired t test, p = 0.01; Figure 1B), suggesting that with the amount of training used in this study the performance is controlled by the action-outcome instrumental contingency.

### Electrode Placement

In 5 rats, MD and mPFC were recorded simultaneously, with each array covering both sides of the brain. Three rats were implanted in the MD only. Histological analysis showed clear electrode tracks and recoding sites in MD and mPFC (mainly prelimbic and infralimbic regions), but not in the anterior cingulate cortex (Figure 1A). We recorded from a total of 268 neurons from MD (n = 69, 71, 66, 62 for each recording session) and 170 neurons from mPFC (n = 44, 45, 44, 37 per session). Based on the waveform differences over days from the same electrode, new neurons were considered to be recorded each day.

### Changes in Single Unit Activity during Acquisition

**Figure 2:**
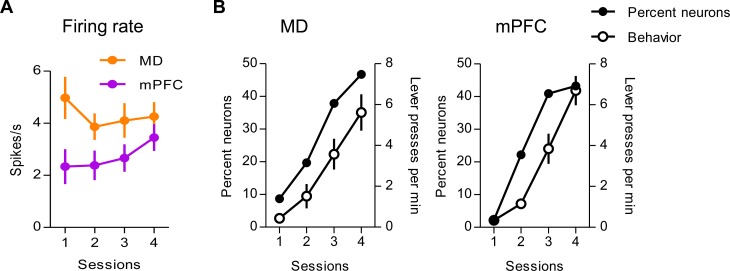
Neural plasticity in MD and mPFC during learning. A, The firing rates of all responsive neurons in the MD (n = 69, 71, 66, 62 for each recording session) and mPFC (n = 44, 45, 44, 37) upon the reward delivery during acquisition. B, Dramatic increase in overall percentage of neurons whose activity is modulated by the reward delivery (both excited and inhibited). The rate of lever pressing across 4 training sessions is also plotted MD and mPFC activity was recorded simultaneously from 5 rats. MD activity only was recorded from 3 rats. Error bars represent SEM.

Single unit neural activity was recorded starting with the 1^st^ session of CRF training. All rats learned to press a lever for food pellets within 4 sessions of training. In the beginning very few neurons were task related. With training, however, the neural activity in both MD and mPFC changed dramatically. The most common type of task related modulation was found in response to reward delivery. Figure 2A shows the dynamic changes of the firing rate of all recorded neurons upon the reward delivery across four consecutive sessions. Interestingly, the firing rates of mPFC neurons increased with learning (one-way ANOVA, Kruskal-Wallis test, p = 0.02), but not those of MD neurons (Kruskal-Wallis test, p = 0.71).

**Figure 3:**
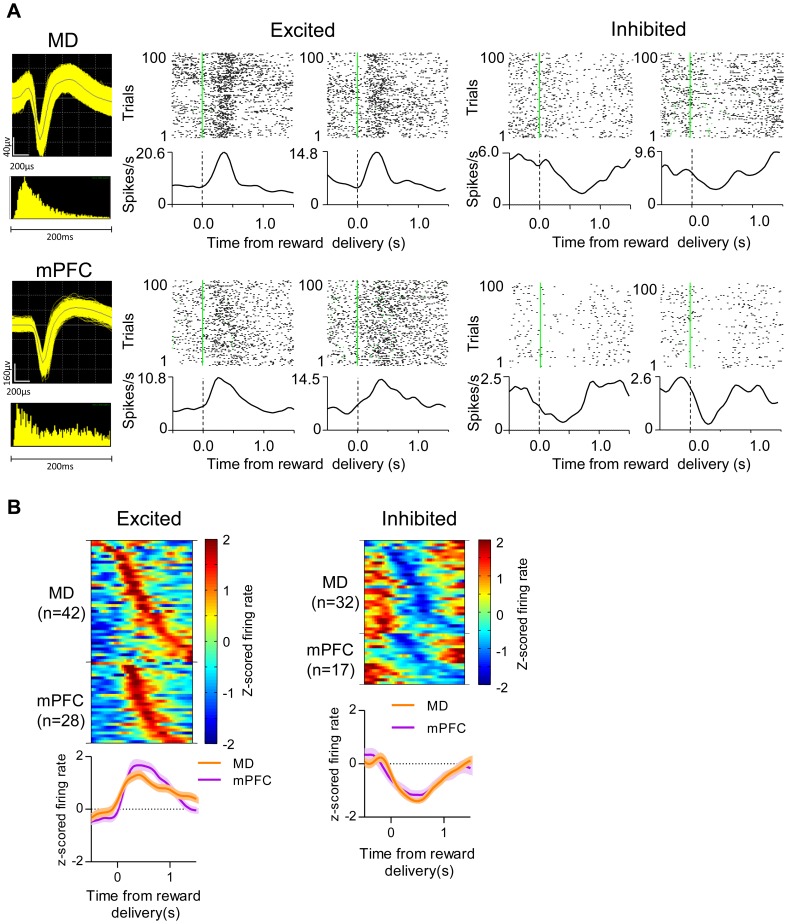
Neuronal activity in MD and mPFC during acquisition. A, Left, action potential waveform and distribution of interspike intervals of representative neurons recorded from MD and mPFC. Right, Perievent raster plots of representative neurons. Each row in raster plot represents a single trial. Green line represents time of reward delivery. Reward excited neuron increases firing after the completion of lever press action and delivery of reward. Reward inhibited neuron decreases firing upon the delivery of reward. B, Top, spike density functions of individual neurons that transiently increased (MD n = 42; mPFC n = 28) or decreased (MD n = 32; mPFC n = 17) activity following reward delivery. Each row shows a z-score normalized spike density function for a single neuron. The neurons are sorted by the latency to the maximum or minimum amplitude. Bottom, normalized population firing rate of reward excited and inhibited neurons at the time of reward delivery. Shaded areas indicate SEM.

**Figure 4:**
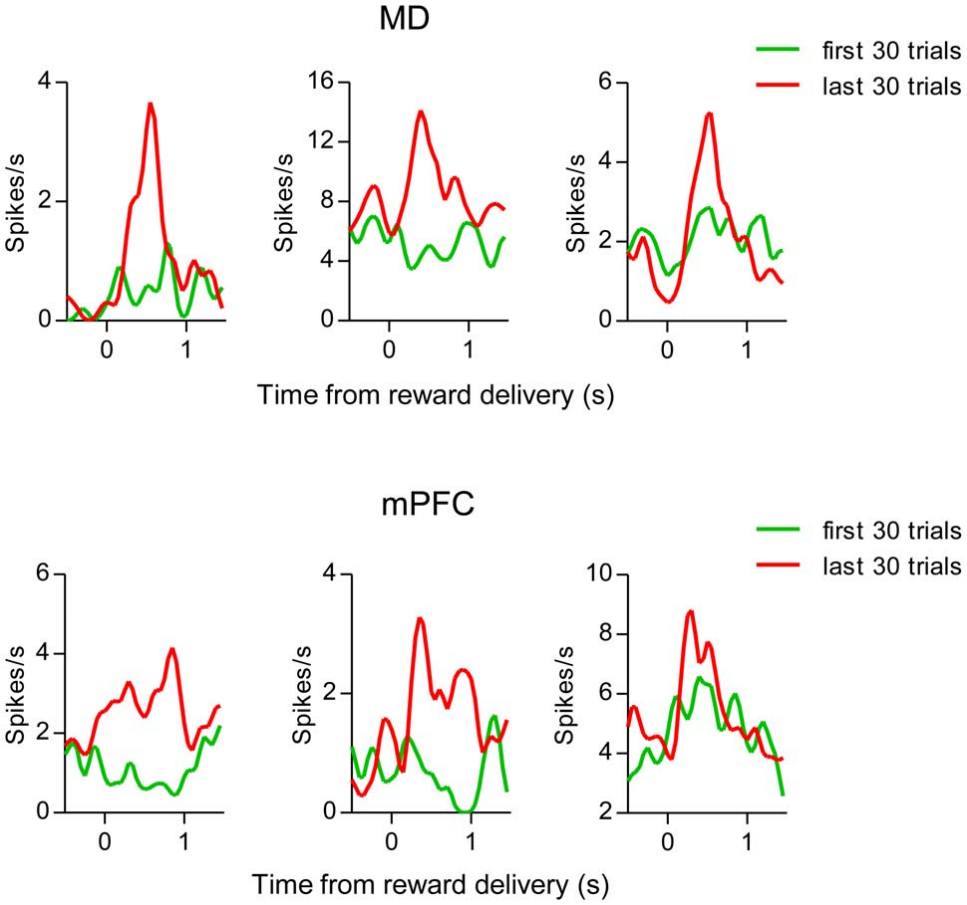
Changes of neuronal responses within a single session. Perievent histogram of representative MD (top) and mPFC (bottom) reward related neurons during the first 30 presses (green) and last 30 presses (red) during the second or third acquisition session.

In both regions, many units responded after the termination of lever press and the delivery of the reward (Figure 2B). The reported increase was observed even when only the first 30 presses from each session were analyzed. Thus, the increased number of lever presses in the later sessions was not responsible for producing this effect. Representative waveforms of the single units are shown in Figure 3A. We found 42 MD neurons and 28 mPFC neurons that were significantly excited by the reward delivery. On the other hand, there were 32 MD neurons and 17 mPFC neurons that reduced firing after reward delivery (Figure 3*B*). Some neurons exhibited clear increased responses to reward delivery even within a single session after learning (Figure 4).

We also analyzed single unit activity just before the lever press. We found that the activity of fewer neurons was modulated by action preparation and initiation. We found 15 "excited" neurons in the MD and 3 in the mPFC; and 17 "inhibited" neurons in the MD and 10 in the mPFC.

### Changes in LFP during Learning

**Figure 5:**
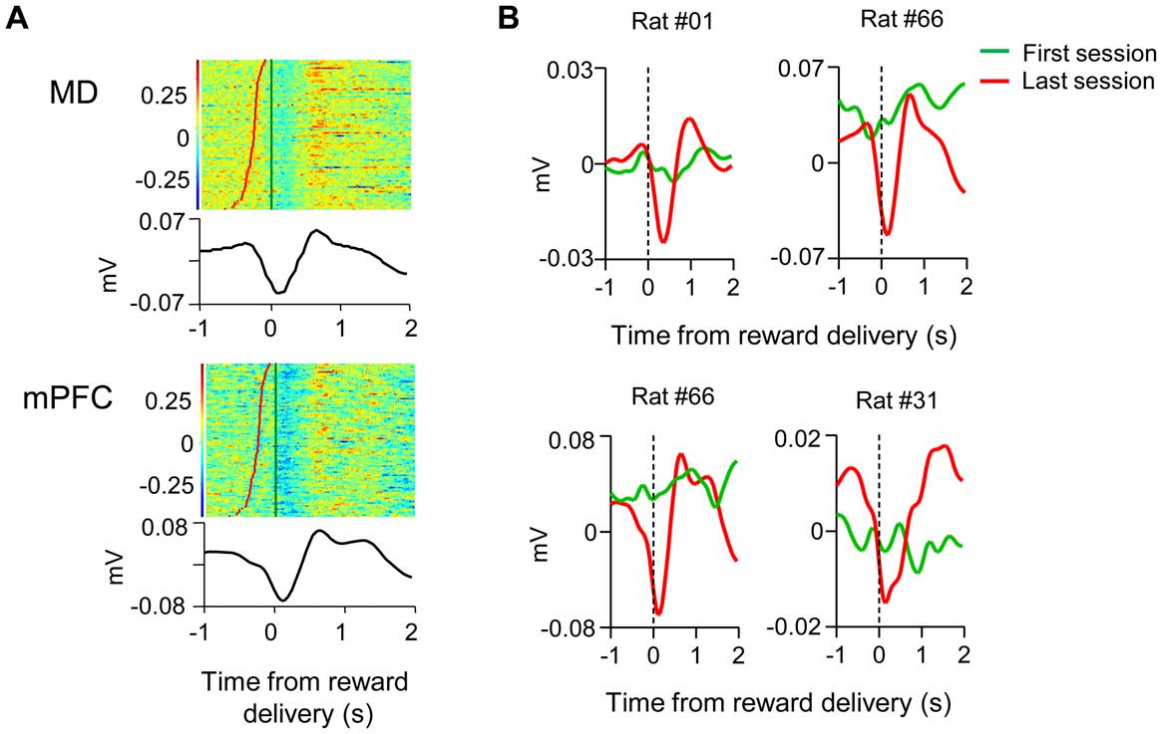
Learning-related modulation in LFP activities. **A,** Perievent raster plots of representative LFP recording. Both examples display depolarization following reward delivery. B, Perivent histograms of representative LFP recorded from 3 rats during the first training session and the last session (top, MD; bottom, mPFC). LFPs exhibit depolarization (negativity in the extracellular recording) with learning.

We also examined changes in LFP during learning. We recorded from 14 mPFC channels from 5 rats in which MD and mPFC were simultaneously recorded, and from 22 MD channels from 5 MD-mPFC and 2 MD rats (1 rat was excluded because of excessive noise in the LFP recording). Representative peri-event histograms are displayed in Figure 5A. Upon reward delivery, a prominent dip was observed in the LFP, indicating a net depolarization in the subthreshold activity of the neuronal population. As shown in Figure 5B, this depolarization increased in the course of learning. The effect was observed when we only analyzed the same number of presses from the first session and the last session, to rule out any differences due to the increase in the number of presses during learning.

### Dynamic Changes in Neural Oscillations Associated with Learning

**Figure 6:**
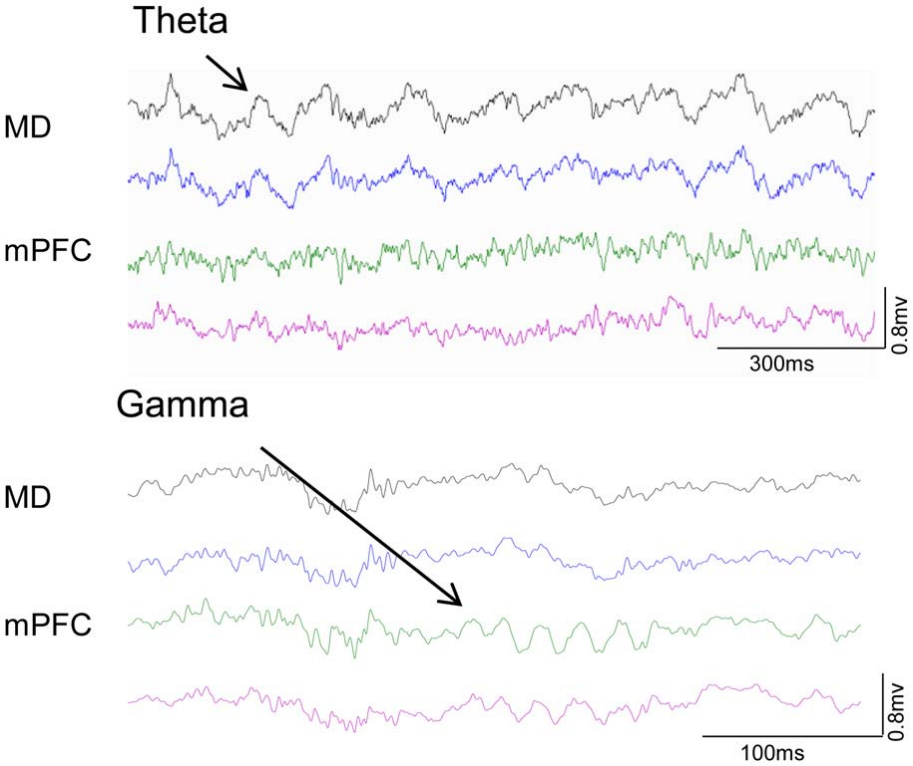
LFP recording in MD and mPFC during behavior. Representative LFP traces recorded from the four electrodes during the final session. LFPs in the MD exhibit prominent theta band (∼7–8 Hz) oscillations, whereas LFPs in the mPFC show prominent gamma band (∼50 Hz) oscillations.

**Figure 7:**
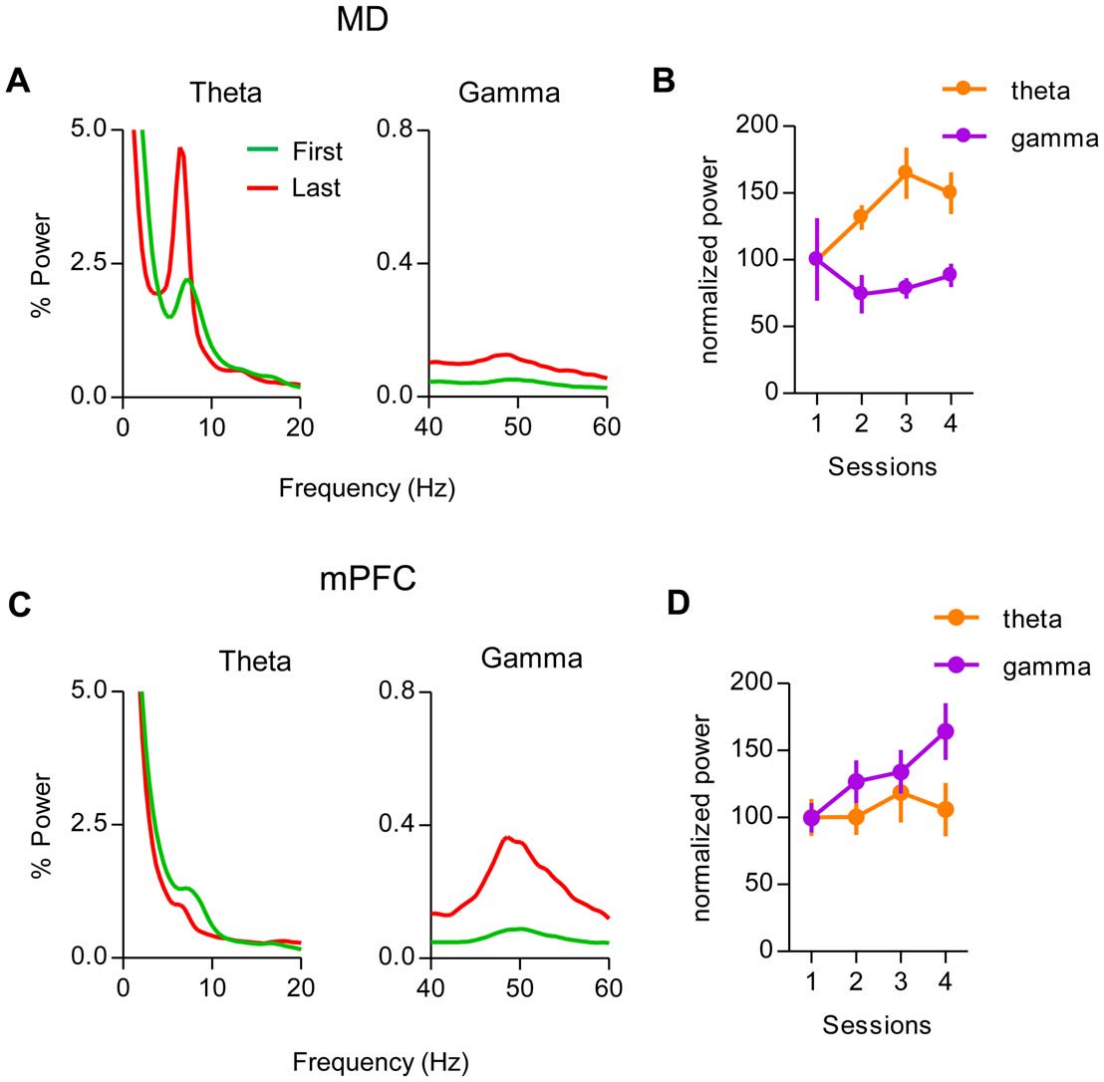
Dynamic changes in oscillatory activity during learning. A, Power spectral analysis of theta and gamma oscillations in the MD. Theta band oscillations increased during training, but gamma oscillations did not. First, first session; Last, last (4th) training session. Representative data are shown from one rat with simultaneous MD and mPFC recordings. B, Normalized (% of the first session) power of theta and gamma oscillations in the MD (n = 22) during acquisition. Theta oscillations in the MD increased significantly over time, whereas gamma oscillations did not. Data from all animals are averaged and shown here. Error bars present SEM. C, Power spectral analysis of theta and gamma oscillations in the mPFC. Representative data are shown from one rat with simultaneous MD and mPFC recordings. D, Normalized (% of the first session) power of theta and gamma oscillations in the mPFC (n = 14) during acquisition. There was a significant increase in the gamma oscillation but not in theta oscillations.

In the MD, LFP showed strong theta oscillations (∼7–8 Hz) and weak gamma oscillations (∼50 Hz), whereas mPFC LFP showed the opposite pattern (Figure 6). More importantly, as shown in Figure 7, the overall oscillatory activity in both MD and mPFC changed dramatically during learning. In the MD, theta power increased during learning (Figure 7B, one-way ANOVA, F = 5.75, p = 0.002), but gamma power did not change significantly (F = 0.53, p = 0.66). In the mPFC, on the other hand, gamma oscillations became very pronounced after learning (Figure 7D, one-way ANOVA, F = 4.60, p = 0.008), but no significant changes were seen in the theta power (F = 1.15, p = 0.34).

**Figure 8:**
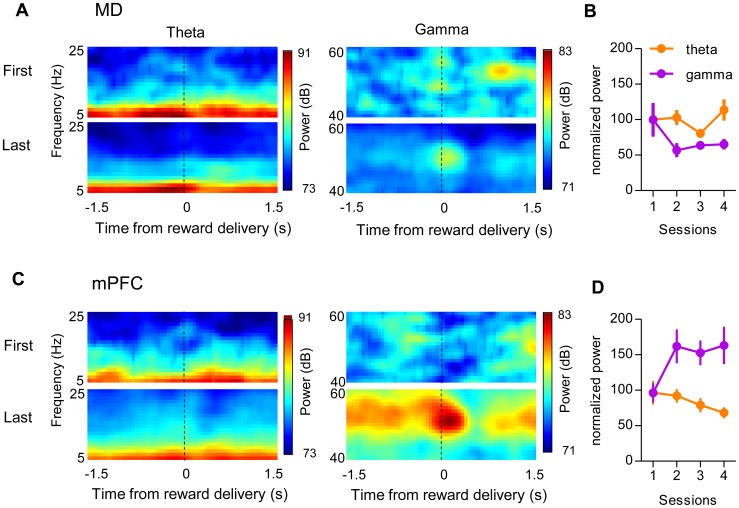
Theta and gamma frequency oscillations. (A and C) Perievent spectrograms of representative MD (*A*) and mPFC *(C)* LFP during the first (top) and last (bottom) session. MD Theta power is much stronger compare to mPFC. mPFC gamma power is much stronger compared to MD. After learning, gamma power is maximal at the time of reward delivery. (B and D) Changes of normalized power spectra of theta and gamma frequency oscillations in MD (n = 22) *(B)* and mPFC (n = 14) *(D*) upon the reward deliver across four sessions. Error bars indicate SEM.

In accord with our single unit recording data, we did not find significant modulation of the LFP during the action initiation period (just before the lever press). But gamma power in both MD and mPFC peaked upon the reward delivery. mPFC showed higher gamma power compared to MD. Two representative peri-event spectrograms are shown in Figure 8. LFP oscillations upon reward delivery (during the time window from the reward delivery to the start of the head entry into the food cup) in both MD and mPFC changed differentially across training sessions. In the MD, neither theta nor gamma power changed significantly during acquisition (repeated measures ANOVA, Fs <2.33, ps>0.05). By contrast, in the mPFC, gamma oscillations became more pronounced with training (repeated measures ANOVA, F = 3.21, p = 0.03). Interestingly, theta oscillations upon reward delivery were reduced (repeated measures ANOVA, F = 3.23, p = 0.03). These findings suggested that theta and gamma oscillations were differentially modulated throughout the training sessions.

### Changes in Coherence between MD and mPFC Activity during Learning

**Figure 9:**
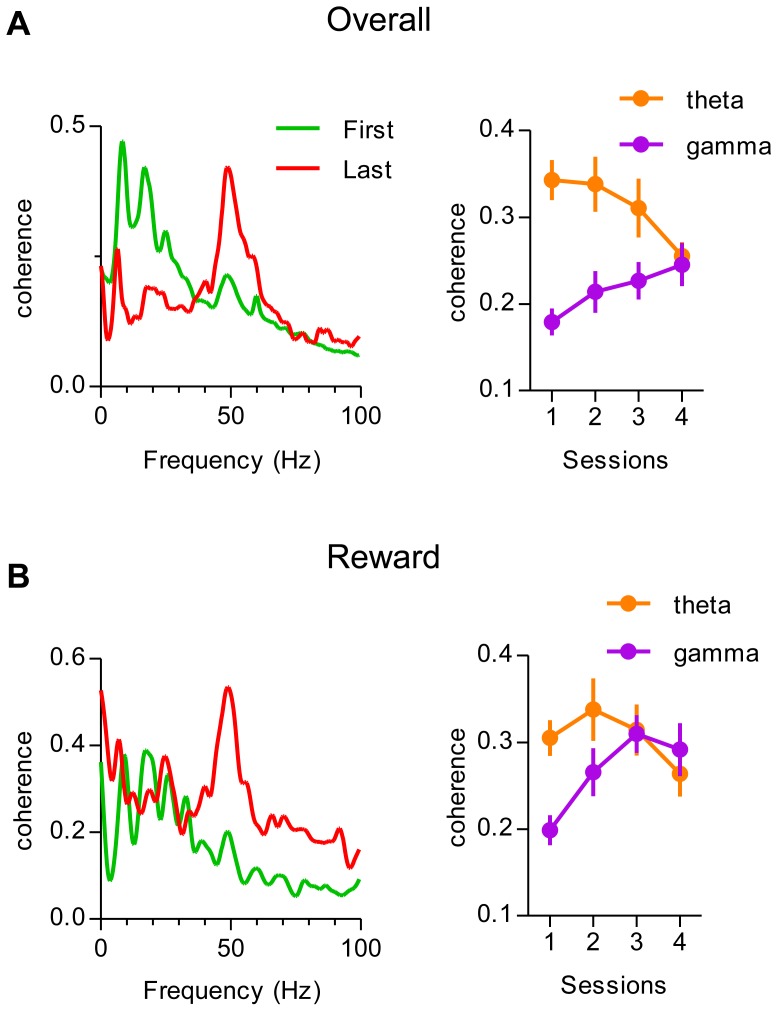
Changes in coherence between MD and mPFC during learning. A, Left, Overall coherence from two representative electrodes during the first (green) and last (red) session in MD and mPFC. Right, dynamic changes of theta and gamma coherence during 4 sessions of instrumental learning (n = 33 pairs in each session). Error bars represent SEM. B, Left, coherence upon reward delivery measured from activity from two representative electrodes. Right, dynamic modulation of theta and gamma coherence by the reward delivery. Error bars indicate SEM.

To determine the dynamic interactions between MD and mPFC during learning, we analyzed the coherence between these areas across four sessions. Coherence can be used as an estimate of the strength of coupling between activities from two different brain regions. As shown in Figure 9, the overall coherence between MD and mPFC changed significantly during the course of learning. Theta coherence did not change significantly across sessions (repeated measures ANOVA, F = 2.40, p = 0.07). By contrast, gamma coherence was weak at first, but increased significantly with learning (repeated measures ANOVA, F = 3.39, p = 0.02). Next, we examine how the coherence between MD and mPFC was modulated by reward delivery across sessions. Gamma coherence upon reward delivery increased during learning (repeated measures ANOVA, F = 7.75, p = 0.0001), but theta coherence did not (F = 1.43, p = 0.24).

## Discussion

To understand the role of MD and mPFC in the acquisition of goal directed behavior, we recorded from both areas as rats learned to press a lever for food rewards. All rats learned to press the lever by the end of the first session, and progressively increased their rate of lever pressing (Figure 2). They were able to learn rapidly the relationship between the lever press and reward. Neural activity in this thalamocortical circuit changed dramatically during instrumental learning. Our results suggest that MD and mPFC form a functional circuit, with similar task-related activity which emerges in the course of learning. However, we also found significant differences in the pattern of oscillatory activity in these two regions, and above all in the dynamic changes of such activity during training. Such oscillatory activity was modulated by reward delivery. The coherence between MD and mPFC activity also changed significantly during the course of learning (Table 1).

**Table 1:** Summary of the changes in neural activity and LFP oscillation as result of learning.

Changes		MD	PFC
Neuronalactivity	firing rate	no change	increased
	within session activity	increased	increased
	# of excited neurons	42/268	28/170
	# of inhibited neurons	32/268	17/170
	percent neurons acrosstraining sessions	increased	increased
LFP power	overall theta	increased	no change
	overall gamma	no change	increased
	theta to reward	no change	no change
	gamma to reward	decreased	increased
Coherence	theta (overall & reward)	no change	
	gamma (overall & reward)	increased	

In our study, we recorded from completely naive rats learning to press the lever for the first time. We were thus able to collect data on how neural activity changed during the initial phase of instrumental learning, when the animal rapidly acquired the relationship between the lever press and reward delivery. It is important to note that performance of the action after initial acquisition is highly sensitive to changes in outcome value, as shown by our devaluation test. The lever pressing was therefore clearly goal-directed. The observed plasticity accompanies the acquisition of the action-outcome contingency.

At the start of training, there was virtually no task related neurons in either MD or mPFC. However, as the rats learned to press the lever, many neurons in both regions increased or decreased their rate of firing in relation to lever pressing and reward (Figure 2). The LFP data (Figure 5), which show significant depolarization in the subthreshold activity in response to reward delivery, also suggest that the emergence of reward elicited activity is a widespread phenomenon. To our knowledge, this is the first report of significant plasticity *in vivo* in this thalamocortical circuit during instrumental learning.

For the continuous reinforcement task used in this study, reward is delivered immediately upon the completion of the lever press. Surprisingly, although the firing rate of some neurons were modulated during the action initiation period (starting at 500 ms before the lever press), such neurons are rare in both MD and mPFC. Nor did we observe significant population activity (LFP) that was modulated by action initiation. In contrast, neurons that altered their firing activity following the completion of the action and the reward delivery were much more common, confirmed by the LFP recordings (Figures 3 and 5). These results suggest that the primary role of the MD-mPFC circuit is to signal the outcome of the goal directed behavior, in this case the reward feedback. This is in accord with previous work that learning of stimulus reward associations also requires the MD [21], [22], [23].

### Changes in Oscillatory Activity in Local Field Potential Recording

Oscillatory activities in different frequency ranges are widely found in different brain areas and correlated with behavioral states [37], [38], [39], [40], [41]. Previous work has shown significant changes in oscillations during learning [41], [42], [43], [44]. Despite the similarities between MD and mPFC in their overall pattern of task-related activity, we observed striking differences between these areas in the dynamic changes in oscillatory LFP activity. Above all, gamma power increased in mPFC, but not in MD (Figure 8).

Theta (6–10 Hz) oscillations are common in the prefrontal cortex and hippocampus, often found during exploration and learning [42], [45], [46]. Gamma (40–55 Hz) oscillations, on the other hand, have traditionally been linked to attention [44]. More recently, recording from the rat ventral striatum, Redish and colleagues found that gamma oscillations increased following the delivery of rewards and gamma power increased with learning on a maze task [40], [47]. Both our data and previous work from Redish and colleagues show reward-elicited increase in oscillations in the low gamma range of roughly 50 Hz. Since the mPFC sends excitatory projections directly to the ventral striatum, the reward-elicited increase in gamma power observed in the ventral striatum could in part be caused by this prominent corticostriatal projection.

Theta oscillations has been also shown to working memory performance in rodents [48], monkeys [49] and humans [50]. Gamma oscillations in the PFC are hypothesized to play an important role in attention by enhancing the neuronal representation of attended sensory input and by regulating the communication among neuronal groups in distinct areas that convey the behaviorally relevant information [46].

The coherence measure could reflect the functional interactions between different brain regions [51], [52]. When we measured the overall coherence between simultaneously recorded MD and mPFC LFP during the course of training, we found that theta coherence did not change, whereas gamma coherence increased with instrumental learning. When we examined the coherence in response to the reward delivery, we also found a significant increase in gamma coherence, but theta coherence did not change significantly across sessions (Figure 9). The enhanced gamma coherence could reflect excitatory inputs responsible for the increase in firing rate of single units immediately after reward delivery (Figure 3). Thus, an overall increase in gamma coherence between MD and mPFC in response to reward delivery is the most striking change in the LFP during initial acquisition. Such changes can have a major impact on effective communication between these two structures. Whether the increase in gamma coherence we observed reflects increased perceptual attention to essential environmental feedback for goal-directed actions, or plays a more critical role in the generation of the appropriate action, remains to be determined by future studies that manipulate online neural activity directly.

In short, our data revealed that instrumental learning in a standard operant task is accompanied by dramatic changes in coordination of population activity between MD and mPFC. Few neurons in MD and mPFC changed their activity prior to the initiation of action, suggesting that this thalamocortical circuit is not critical for action initiation and selection, in agreement with the effects of lesions to these two areas [4], [28]. On the other hand, basal ganglia lesions are well known to impair action initiation [53]. Given the strong, projections from the mPFC to the ventral and medial striatal regions, signals representing behavioral outcomes (such as reward) could be transmitted to the basal ganglia, which plays an important role in the learning and expression of goal-directed actions [54]. The role of the MD-mPFC circuit therefore appears to be restricted to the signaling of the reward feedback following the action [55]. Our findings are also in agreement with previous lesion studies implicating MD and mPFC in the learning of the action-outcome contingency [4], [56], [57]. It is important to point out that this thalamocortical circuit alone is not sufficient for instrumental learning; a distributed circuit involving additional brain regions in the basal ganglia is needed [28], [54]. The present study therefore merely represents an initial step in elucidating the computational roles of the brain regions that are essential for the acquisition and expression of goal-directed behaviors.

## References

[1] Miller EK (2000) The prefrontal cortex and cognitive control. Nat Rev Neurosci 1: 59–65. 11252769 10.1038/35036228

[2] Matsumoto K, Suzuki W, Tanaka K (2003) Neuronal correlates of goal-based motor selection in the prefrontal cortex. Science 301: 229–232. 12855813 10.1126/science.1084204

[3] Ridderinkhof KR, Ullsperger M, Crone EA, Nieuwenhuis S (2004) The role of the medial frontal cortex in cognitive control. Science 306: 443–447. 15486290 10.1126/science.1100301

[4] Corbit LH, Muir JL, Balleine BW (2003) Lesions of mediodorsal thalamus and anterior thalamic nuclei produce dissociable effects on instrumental conditioning in rats. Eur J Neurosci 18: 1286–1294. 12956727 10.1046/j.1460-9568.2003.02833.x

[5] Goldman-Rakic PS, Porrino LJ (1985) The primate mediodorsal (MD) nucleus and its projection to the frontal lobe. J Comp Neurol 242: 535–560. 2418080 10.1002/cne.902420406

[6] Giguere M, Goldman-Rakic PS (1988) Mediodorsal nucleus: areal, laminar, and tangential distribution of afferents and efferents in the frontal lobe of rhesus monkeys. J Comp Neurol 277: 195–213. 2466057 10.1002/cne.902770204

[7] Groenewegen HJ (1988) Organization of the afferent connections of the mediodorsal thalamic nucleus in the rat, related to the mediodorsal-prefrontal topography. Neuroscience 24: 379–431. 2452377 10.1016/0306-4522(88)90339-9

[8] Ray JP, Price JL (1992) The organization of the thalamocortical connections of the mediodorsal thalamic nucleus in the rat, related to the ventral forebrain-prefrontal cortex topography. J Comp Neurol 323: 167–197. 1401255 10.1002/cne.903230204

[9] Ray JP, Price JL (1993) The organization of projections from the mediodorsal nucleus of the thalamus to orbital and medial prefrontal cortex in macaque monkeys. J Comp Neurol 337: 1–31. 7506270 10.1002/cne.903370102

[10] Pirot S, Jay TM, Glowinski J, Thierry AM (1994) Anatomical and electrophysiological evidence for an excitatory amino acid pathway from the thalamic mediodorsal nucleus to the prefrontal cortex in the rat. Eur J Neurosci 6: 1225–1234. 7524967 10.1111/j.1460-9568.1994.tb00621.x

[11] Kuroda M, Yokofujita J, Murakami K (1998) An ultrastructural study of the neural circuit between the prefrontal cortex and the mediodorsal nucleus of the thalamus. Prog Neurobiol 54: 417–458. 9522395 10.1016/s0301-0082(97)00070-1

[12] Floresco SB, Grace AA (2003) Gating of hippocampal-evoked activity in prefrontal cortical neurons by inputs from the mediodorsal thalamus and ventral tegmental area. J Neurosci 23: 3930–3943. 12736363 10.1523/JNEUROSCI.23-09-03930.2003 PMC6742171

[13] Ferron A, Thierry AM, Le Douarin C, Glowinski J (1984) Inhibitory influence of the mesocortical dopaminergic system on spontaneous activity or excitatory response induced from the thalamic mediodorsal nucleus in the rat medial prefrontal cortex. Brain Res 302: 257–265. 6733513 10.1016/0006-8993(84)90238-5

[14] Herry C, Vouimba RM, Garcia R (1999) Plasticity in the mediodorsal thalamo-prefrontal cortical transmission in behaving mice. J Neurophysiol 82: 2827–2832. 10561450 10.1152/jn.1999.82.5.2827

[15] McDonald AJ (1991) Organization of amygdaloid projections to the prefrontal cortex and associated striatum in the rat. Neuroscience 44: 1–14. 1722886 10.1016/0306-4522(91)90247-l

[16] Maurice N, Deniau JM, Glowinski J, Thierry AM (1998) Relationships between the prefrontal cortex and the basal ganglia in the rat: physiology of the corticosubthalamic circuits. J Neurosci 18: 9539–9546. 9801390 10.1523/JNEUROSCI.18-22-09539.1998 PMC6792878

[17] Maurice N, Deniau JM, Menetrey A, Glowinski J, Thierry AM (1998) Prefrontal cortex-basal ganglia circuits in the rat: involvement of ventral pallidum and subthalamic nucleus. Synapse 29: 363–370. 9661254 10.1002/(SICI)1098-2396(199808)29:4<363::AID-SYN8>3.0.CO;2-3

[18] Yin HH, Knowlton BJ (2006) The role of the basal ganglia in habit formation. Nat Rev Neurosci 7: 464–476. 16715055 10.1038/nrn1919

[19] Haber SN, Calzavara R (2009) The cortico-basal ganglia integrative network: the role of the thalamus. Brain Res Bull 78: 69–74. 18950692 10.1016/j.brainresbull.2008.09.013 PMC4459637

[20] Rushworth MF, Noonan MP, Boorman ED, Walton ME, Behrens TE (2011) Frontal cortex and reward-guided learning and decision-making. Neuron 70: 1054–1069. 21689594 10.1016/j.neuron.2011.05.014

[21] Gaffan D, Murray EA (1990) Amygdalar interaction with the mediodorsal nucleus of the thalamus and the ventromedial prefrontal cortex in stimulus-reward associative learning in the monkey. J Neurosci 10: 3479–3493. 2230939 10.1523/JNEUROSCI.10-11-03479.1990 PMC6570092

[22] Hunt PR, Aggleton JP (1998) Neurotoxic lesions of the dorsomedial thalamus impair the acquisition but not the performance of delayed matching to place by rats: a deficit in shifting response rules. J Neurosci 18: 10045–10052. 9822759 10.1523/JNEUROSCI.18-23-10045.1998 PMC6793303

[23] Mitchell AS, Baxter MG, Gaffan D (2007) Dissociable performance on scene learning and strategy implementation after lesions to magnocellular mediodorsal thalamic nucleus. J Neurosci 27: 11888–11895. 17978029 10.1523/JNEUROSCI.1835-07.2007 PMC2241732

[24] Mitchell AS, Browning PG, Baxter MG (2007) Neurotoxic lesions of the medial mediodorsal nucleus of the thalamus disrupt reinforcer devaluation effects in rhesus monkeys. J Neurosci 27: 11289–11295. 17942723 10.1523/JNEUROSCI.1914-07.2007 PMC2242856

[25] Ostlund SB, Balleine BW (2008) Differential involvement of the basolateral amygdala and mediodorsal thalamus in instrumental action selection. J Neurosci 28: 4398–4405. 18434518 10.1523/JNEUROSCI.5472-07.2008 PMC2652225

[26] Balleine BW, Dickinson A (1998) Goal-directed instrumental action: contingency and incentive learning and their cortical substrates. Neuropharmacology 37: 407–419. 9704982 10.1016/s0028-3908(98)00033-1

[27] Killcross S, Coutureau E (2003) Coordination of actions and habits in the medial prefrontal cortex of rats. Cereb Cortex 13: 400–408. 12631569 10.1093/cercor/13.4.400

[28] Corbit LH, Balleine BW (2003) The role of prelimbic cortex in instrumental conditioning. Behav Brain Res 146: 145–157. 14643467 10.1016/j.bbr.2003.09.023

[29] Haddon JE, Killcross S (2006) Prefrontal cortex lesions disrupt the contextual control of response conflict. J Neurosci 26: 2933–2940. 16540570 10.1523/JNEUROSCI.3243-05.2006 PMC6673980

[30] Hadland KA, Rushworth MF, Gaffan D, Passingham RE (2003) The anterior cingulate and reward-guided selection of actions. J Neurophysiol 89: 1161–1164. 12574489 10.1152/jn.00634.2002

[31] Adams CD, Dickinson A (1981) Instrumental responding following reinforcer devaluation. Quarterly Journal of Experimental Psychology 33: 109–122.

[32] Fan D, Rossi MA, Yin HH (2012) Mechanisms of action selection and timing in substantia nigra neurons. J Neurosci 32: 5534–5548. 22514315 10.1523/JNEUROSCI.5924-11.2012 PMC6703499

[33] Fan D, Rich D, Holtzman T, Ruther P, Dalley JW, et al (2011) A wireless multi-channel recording system for freely behaving mice and rats. PLoS One 6: e22033. 21765934 10.1371/journal.pone.0022033 PMC3134473

[34] Pereda E, Quiroga RQ, Bhattacharya J (2005) Nonlinear multivariate analysis of neurophysiological signals. Prog Neurobiol 77: 1–37. 16289760 10.1016/j.pneurobio.2005.10.003

[35] Sakkalis V, Doru Giurc Neanu C, Xanthopoulos P, Zervakis ME, Tsiaras V, et al (2009) Assessment of linear and nonlinear synchronization measures for analyzing EEG in a mild epileptic paradigm. IEEE Trans Inf Technol Biomed 13: 433–441. 19273019 10.1109/TITB.2008.923141

[36] Dickinson A (1985) Actions and habits: the development of behavioural autonomy. Philosophical Transactions of the Royal Society B308: 67–78.

[37] Buzsaki G, Anastassiou CA, Koch C (2012) The origin of extracellular fields and currents - EEG, ECoG, LFP and spikes. Nat Rev Neurosci 13: 407–420. 22595786 10.1038/nrn3241 PMC4907333

[38] Basar E, Basar-Eroglu C, Karakas S, Schurmann M (2000) Brain oscillations in perception and memory. Int J Psychophysiol 35: 95–124. 10677641 10.1016/s0167-8760(99)00047-1

[39] Buzsaki G, Wang XJ (2012) Mechanisms of Gamma Oscillations. Annu Rev Neurosci. 10.1146/annurev-neuro-062111-150444 PMC4049541 22443509

[40] van der Meer MA, Redish AD (2009) Low and High Gamma Oscillations in Rat Ventral Striatum have Distinct Relationships to Behavior, Reward, and Spiking Activity on a Learned Spatial Decision Task. Front Integr Neurosci 3: 9. 19562092 10.3389/neuro.07.009.2009 PMC2701683

[41] Berke JD, Okatan M, Skurski J, Eichenbaum HB (2004) Oscillatory entrainment of striatal neurons in freely moving rats. Neuron 43: 883–896. 15363398 10.1016/j.neuron.2004.08.035

[42] DeCoteau WE, Thorn C, Gibson DJ, Courtemanche R, Mitra P, et al (2007) Learning-related coordination of striatal and hippocampal theta rhythms during acquisition of a procedural maze task. Proc Natl Acad Sci U S A 104: 5644–5649. 17372196 10.1073/pnas.0700818104 PMC1838454

[43] Benchenane K, Peyrache A, Khamassi M, Tierney PL, Gioanni Y, et al (2010) Coherent theta oscillations and reorganization of spike timing in the hippocampal- prefrontal network upon learning. Neuron 66: 921–936. 20620877 10.1016/j.neuron.2010.05.013

[44] Fries P, Reynolds JH, Rorie AE, Desimone R (2001) Modulation of oscillatory neuronal synchronization by selective visual attention. Science 291: 1560–1563. 11222864 10.1126/science.1055465

[45] Hasselmo ME (2005) What is the function of hippocampal theta rhythm?–Linking behavioral data to phasic properties of field potential and unit recording data. Hippocampus 15: 936–949. 16158423 10.1002/hipo.20116

[46] Benchenane K, Tiesinga PH, Battaglia FP (2011) Oscillations in the prefrontal cortex: a gateway to memory and attention. Curr Opin Neurobiol 21: 475–485. 21429736 10.1016/j.conb.2011.01.004

[47] van der Meer MA, Redish AD (2009) Covert Expectation-of-Reward in Rat Ventral Striatum at Decision Points. Front Integr Neurosci 3: 1. 19225578 10.3389/neuro.07.001.2009 PMC2644619

[48] Jones MW, Wilson MA (2005) Theta rhythms coordinate hippocampal-prefrontal interactions in a spatial memory task. PLoS Biol 3: e402. 16279838 10.1371/journal.pbio.0030402 PMC1283536

[49] Lee H, Simpson GV, Logothetis NK, Rainer G (2005) Phase locking of single neuron activity to theta oscillations during working memory in monkey extrastriate visual cortex. Neuron 45: 147–156. 15629709 10.1016/j.neuron.2004.12.025

[50] Cashdollar N, Malecki U, Rugg-Gunn FJ, Duncan JS, Lavie N, et al (2009) Hippocampus-dependent and -independent theta-networks of active maintenance. Proc Natl Acad Sci U S A 106: 20493–20498. 19918077 10.1073/pnas.0904823106 PMC2787111

[51] Singer W (1999) Neuronal synchrony: a versatile code for the definition of relations? Neuron 24: 49–65, 111–125. 10.1016/s0896-6273(00)80821-1 10677026

[52] Varela F, Lachaux JP, Rodriguez E, Martinerie J (2001) The brainweb: phase synchronization and large-scale integration. Nat Rev Neurosci 2: 229–239. 11283746 10.1038/35067550

[53] Grillner S, Hellgren J, Menard A, Saitoh K, Wikstrom MA (2005) Mechanisms for selection of basic motor programs - roles for the striatum and pallidum. Trends Neurosci. 10.1016/j.tins.2005.05.004 15935487

[54] Yin HH, Ostlund SB, Knowlton BJ, Balleine BW (2005) The role of the dorsomedial striatum in instrumental conditioning. Eur J Neurosci 22: 513–523. 16045504 10.1111/j.1460-9568.2005.04218.x

[55] Luk CH, Wallis JD (2009) Dynamic encoding of responses and outcomes by neurons in medial prefrontal cortex. J Neurosci 29: 7526–7539. 19515921 10.1523/JNEUROSCI.0386-09.2009 PMC2718717

[56] Yu C, Gupta J, Yin HH (2010) The role of mediodorsal thalamus in temporal differentiation of reward-guided actions. Front Integr Neurosci 4. 10.3389/fnint.2010.00014 PMC2901151 20725508

[57] Ostlund SB, Balleine BW (2005) Lesions of medial prefrontal cortex disrupt the acquisition but not the expression of goal-directed learning. J Neurosci 25: 7763–7770. 16120777 10.1523/JNEUROSCI.1921-05.2005 PMC6725247

[58] Paxinos G, Watson C (1998) The rat brain in sterotaxic coordinates. San Diego: Academic Press.

